# Predicting two-year survival versus non-survival after first myocardial infarction using machine learning and Swedish national register data

**DOI:** 10.1186/s12911-017-0500-y

**Published:** 2017-07-05

**Authors:** John Wallert, Mattia Tomasoni, Guy Madison, Claes Held

**Affiliations:** 1 Department of Public Health and Caring Sciences, Uppsala University, Box 564, Husargatan 3, SE - 75122 Uppsala, Sweden; 2 Department of Women’s and Children’s Health, Uppsala University, Box 572, Husargatan 3, SE - 75123 Uppsala, Sweden; 3 Department of Psychology, Umeå University, Hus Y, Behavioral Sciences Building, Vindarnas Torg, Mediagränd 14 B-115, 901 87 Umeå, Sweden; 4 Department of Medical Sciences, Uppsala University, Uppsala, Sweden; 5 Uppsala Clinical Research Center, Uppsala University, Dag Hammarskölds väg 50 A, Uppsala Science Park, 751 83 Uppsala, Sweden

**Keywords:** Cardiovascular disease, Classification, Coronary Artery Syndrome, Prognostic Modelling, Myocardial infarction, Registries, Supervised machine learning

## Abstract

**Background:**

Machine learning algorithms hold potential for improved prediction of all-cause mortality in cardiovascular patients, yet have not previously been developed with high-quality population data. This study compared four popular machine learning algorithms trained on unselected, nation-wide population data from Sweden to solve the binary classification problem of predicting survival versus non-survival 2 years after first myocardial infarction (MI).

**Methods:**

This prospective national registry study for prognostic accuracy validation of predictive models used data from 51,943 complete first MI cases as registered during 6 years (2006–2011) in the national quality register SWEDEHEART/RIKS-HIA (90% coverage of all MIs in Sweden) with follow-up in the Cause of Death register (> 99% coverage). Primary outcome was AUROC (*C*-statistic) performance of each model on the untouched test set (40% of cases) after model development on the training set (60% of cases) with the full (39) predictor set. Model AUROCs were bootstrapped and compared, correcting the *P*-values for multiple comparisons with the Bonferroni method. Secondary outcomes were derived when varying sample size (1–100% of total) and predictor sets (39, 10, and 5) for each model. Analyses were repeated on 79,869 completed cases after multivariable imputation of predictors.

**Results:**

A Support Vector Machine with a radial basis kernel developed on 39 predictors had the highest complete cases performance on the test set (AUROC = 0.845, PPV = 0.280, NPV = 0.966) outperforming Boosted C5.0 (0.845 vs. 0.841, *P* = 0.028) but not significantly higher than Logistic Regression or Random Forest. Models converged to the point of algorithm indifference with increased sample size and predictors. Using the top five predictors also produced good classifiers. Imputed analyses had slightly higher performance.

**Conclusions:**

Improved mortality prediction at hospital discharge after first MI is important for identifying high-risk individuals eligible for intensified treatment and care. All models performed accurately and similarly and because of the superior national coverage, the best model can potentially be used to better differentiate new patients, allowing for improved targeting of limited resources. Future research should focus on further model development and investigate possibilities for implementation.

**Electronic supplementary material:**

The online version of this article (doi:10.1186/s12911-017-0500-y) contains supplementary material, which is available to authorized users.

## Background

Myocardial Infarction (MI) is an acute manifestation of cardiovascular disease (CVD) globally afflicting more than 7 million people annually. Most of the MI risk is ascribed so-called modifiable risk factors, e.g. hyperlipidaemia, diabetes mellitus, smoking, physical inactivity, and obesity. The major underlying cause of MI is coronary heart disease (CHD) [1]. CHD was the leading cause of death worldwide in 2013 (8.14 million deaths, 17% of total), a substantial increase from year 1990 (5.74 million, 12% of total) [2, 3]. Suffering a first MI increases the risk of death and these patients should be monitored closely [4]. If the mortality risk is accurately classified already at hospital discharge this might lead to improved tailoring and efficiency of secondary prevention. Furthermore, population ageing is a growing health concern in most countries, [5] and age is the single most important risk factor for CHD. As the proportion of first MI patients that are middle-aged or older increases, [6] improved care for these patients constitutes a greater net benefit.

Capitalizing on advances within the field of Machine Learning (ML) [7] might improve mortality prognostics in first MI patients. In recent years, ML models have accurately classified complex pathology and intervention outcomes. Examples involve cancer, [8] Alzheimer’s disease, [9] and stroke [10]. Multivariate classification modelling using different ML algorithms is insufficiently researched within cardiology and may complement already established risk estimation tools, such as GRACE [11]. To date, ML has predominantly been applied to narrow and small cardiovascular datasets. One rare exception is a large CVD risk prediction study using ML modelling developed with electronic health record data, however also limited to a selected military veteran subpopulation [12]. To the extent of our knowledge, unselected population data from national high-quality cardiovascular registers have yet not been applied to develop and evaluate ML prediction models. If accurate, the superior generalizability of such models should render them particularly suited for national implementation.

The aim of the present study was to use 6 years of Swedish real-world population data gathered at the time of acute admission *and* treatment for first MI to construct and evaluate four different algorithms predicting all-cause mortality 2 years later. Crucial for this study was the Swedish national quality register on cardiovascular disease, SWEDEHEART [13]. We evaluated (1) boosted C5.0 trees/rule-sets (C5.0), (2) Random Forests (RF), and (3) Support Vector Machines (SVM), and benchmarked these more recently developed algorithms performance against (4) “classic” Logistic regression (LR). LR is a well-established procedure [14] widely used for predictive modelling, including cardiovascular disease [15]. We hypothesized that all models would predict two-year mortality in first MI patients with a concordance statistic high enough to hold clinical potential (Area Under the Receiver Operating Characteristics Curve (AUROC) > 0.70), and further hypothesized that classification performance would decrease independently as (a) the number of cases was reduced from 100% to 1%, and (b) less important predictors were removed from the full predictor set.

## Methods

### Register data

As part of SWEDEHEART, the national quality Register for Information and Knowledge about Swedish Heart Intensive Care Admissions (RIKS-HIA) prospectively registers patients admitted to all Coronary Care Units (CCU) in Sweden for symptoms of acute coronary syndrome. More than 100 variables are collected, and the randomly audited and regularly monitored RIKS-HIA provides excellent coverage of the Swedish population (~ 90% of all MIs < 80 years of age). Independently of the present study, MI diagnosis according to ICD codes I21-I23 [16] was decided by the hospital cardiologist based on clinical symptoms, electrocardiogram, and additional information. RIKS-HIA provided data on 156,690 MIs suffered by 135,934 patients between January 1st 2006 and December 31st 2013. RIKS-HIA is approved by the Swedish Data Inspection Board, and the National Board of Health and Welfare [13]. Through personal identification number linkage, the Cause of Death register supplied death dates for patients that died during the study period with >99% population coverage [17].

### Predictor pre-processing

Chief cardiologist and SWEDEHEART register data expertise (CH) was used to reduce the number of predictors from >100 to 69. This initial feature selection was inclusive, meaning that any type of predictors that would possibly indicate future mortality were kept. Thus, redundant proxies and other known nuisance variables were removed in this initial stage. Remaining predictors were deliberately heterogeneous, including established mortality risk indicators (e.g. comorbid diabetes), important survival factors (e.g. statin treatment), of immutable (e.g. age) and modifiable (e.g. smoking) origin. Predictors considered less important for the outcome and with >5% missing values (*N* = 8) were removed. Predictors considered more important and with >15% missing values (*N* = 5) were also removed. Multicollinearity was a non-issue as continuous predictor correlations were low (Pearson r range = −.36 to .09). One-of-k coding was used. To ensure predictor representation within single resampling folds, near-zero variance predictors were removed (*N* = 17). This rendered a *full predictor set* of 39 variables (5 continuous, 34 dichotomous). Although routinely registered, it can be unfeasible to collect 39 predictor values for one patient. A *reduced predictor set* (*N* = 10) and a *minimal predictor set* (*N* = 5) were therefore also constructed and evaluated. For such dimensionality reduction, the default predictor rankings for each model selected predictors for the reduced and minimal predictor sets. In this way, some models selected different variables for the 10 and 5 predictor subsets using different selection criteria. A prerequisite for model comparison was that each model had the same full predictor set and patient cases to select from. The predictor selection algorithm BORUTA [18] was investigated but deemed unsuitable because it selects predictors biased towards random forest models, making model comparisons difficult to interpret.

### Classification and sample pre-processing

We ran the first MI date together with the death register to determine which patients were still alive (survivor) or deceased (non-survivor) 2 years later. Cases not part of the studied population (recurrent MIs) and cases lacking adequate exposure time for the outcome in the dataset (registered after the 31st of December 2011 in RIKS-HIA) were excluded (32,430 cases). Also excluded were 22,790 cases of first MI that occurred before the data extraction period (marked as “Yes” on the variable “Previous MI”) and 845 unsure cases in this regard. Because some models and modelling steps required complete data, an additional 27,926 incomplete cases were set aside, yielding a primary sample of 51,943 (5710 deceased) first MI patients with no missing values in the full predictor set. For investigating the effect of sample size on classification performance, outcome stratified random sampling was applied to generate subsamples down to 1% of total cases keeping the class proportions constant. Secondary analyses were conducted on 79,869 cases after multivariable imputation through chained equations and predictive mean matching [19]. For each variable, this method imputes missing values with real values borrowed from other cases which predicted values are closest. The maximum number of multiple imputations was set to 5. All but five predictors had less than 5% missing values before imputation, and those were Weight (10.8%), Smoking (8.4%), Troponin (7.0%), Atrial fibrillation at CCU discharge (6.9%), and Systolic Blood Pressure (6.3%).

### Pseudo-randomisation and data partitioning

All stochastic computer operations were initiated with a constant starting seed, which had the result that modelling steps were reproducible and models directly comparable, since the same cases were selected in the resampling for different models. We applied stratified random splits of data [20] with 60% used for model training and 40% for testing.

### Model tuning, training, and testing

To counter overfitting and achieve robust results, adaptive 7-fold cross-validation resampling with 3 repeats was used for model development on the training set. Within this resampling, we applied (1) a tune-grid search of length 15, (2) random down-sampling of the majority class, and (3) predictor centring and scaling. Grid search evaluates k length of evenly increasing values of any model tuning parameter(s). It then selects the parameter value(s) with the highest performance on the training set and uses this setting to construct the final model on the full training set used for later testing [20, 21]. We down-sampled because a large majority of first MI patients are still alive 2 years later. Since classes were heavily unbalanced, we also tuned, trained, and tested models on the performance metric AUROC. AUROC is a single, rank discrimination statistic that is insensitive to class imbalances. Calculated via the trapezoidal rule, AUROC is the area under the resulting curve when plotting a binary classifier’s true positive rate (sensitivity) as a function of its false positive rate (1 – specificity) for all possible cut-off thresholds [22]. AUROC values range between 1 (a perfect classifier) and 0 (a perfect classifier if inverted) where 0.5 corresponds to random guessing (a useless classifier). AUROC >0.7 might be considered a lower threshold for a potentially useful clinical classifier although this is a much more complex judgement also based on the base rate incidence, consequences of false negatives/positives, test risk, monetary cost, and more. Accuracy was inappropriate as performance metric since optimizing models on Accuracy with heavily unbalanced classes biases models towards predicting all cases as belonging to the majority class. Since we optimized on AUROC, models assigned more error-weight to false negatives than false positives. False negatives are also reasonably considered more costly than false positives for the present mortality prediction. Further specification of such weighting should be tailored to the clinical situation, which is beyond the scope of this paper. The hold-out, untouched test set was only used for validation, i.e. the final performance test of developed models. This untouched set was not down-sampled. Instead it was predicted according to the class incidence as occurring in the clinical population.

### Algorithms

A brief description of the four employed algorithms follows. Further details are available in the Additional file 1.

Binomial LR is a linear model that assumes a Bernoulli distribution of the outcome and a log-linear relationship with the predictors [14]. LR predicts the binary response probability for the outcome class given the predictor values. In contrast with the three subsequent algorithms, LR lacks tuning parameters. The magnitude of the z-values from the LR was used as predictor importance rank.

Boosted C5.0 is a non-linear model that constructs an ensemble of decision trees from multiple single trees in a stage-wise procedure, up-weighting previously misclassified cases through adaptive boosting [23, 24]. A tree splits data at binary decision nodes, recursively dividing the preceding data into two branches. For each tree at each decision node split, C5.0 selects the optimal variable and variable cut-off value so that entropy reduction is maximized. The tree evolves in this manner until it is ended in terminal nodes. Pessimistic pruning reduces the tree complexity [25]. The C5.0 trees then majority votes on the outcome class of a new case. The portion of total cases that fall in terminal nodes after a predictor split determined the C5.0 predictor rank.

The RF is a non-linear model that constructs an ensemble of decision trees. We used the RF version which combines bootstrap sampling of data for constructing each tree, and random subselection of predictors at each decision node [26, 27]. The RF trees majority votes on outcome class. The RF predictor rank was determined by the Gini importance index, i.e. the reduction in node impurity across trees. Thus, a predictor chosen as root split for many trees gets a higher Gini importance than a predictor chosen less frequently and/or for descendant nodes.

The non-linear SVM projects data into a multidimensional hyperspace, in which each case is mapped as a vector. A hyperplane is fitted to data so that the margin between the classes is maximized using the support vectors, i.e. the closest cases with opposite class labels. We selected the radial basis function as kernel for the present soft-margin SVM, [28, 29] respectively allowing for non-linear classification and some overlap between classes. The SVM output was scaled to make the classifier probabilistic, using Platt’s scaling. The AUROC value for each single predictor when separately modelled on the outcome determined the predictor importance rank. This differs from the preceding algorithms, which instead ranked each predictor relative to the other predictors in the model.

### Additional statistics

We present Gaussian continuous variables as mean ± SD, non-Gaussian continuous variables as median (IQR), and categorical variables as count (%). For univariate class comparisons, Welch’s t-test for Gaussian continuous predictors, Mann-Whitney’s *U*-test for non-Gaussian continuous predictors, and Pearson’s χ^2^-test for categorical predictors were used. For the main comparison of model performances, we present Bonferroni-corrected pairwise D-tests (bootstrapped *n* = 10,000) on model AUROCs for each predictor set. Statistical significance was set to 5% (two-tailed).

### Software

We used custom software developed in C# to select patients and code variables. Analyses were performed in R (version 3.2.3, R Development Core Team, Austria, Vienna) with packages *base, C5.0*, *caret, kernlab, mice, plyr, pROC, randomForest, and stats.* [19, 24, 30–34].

## Results

Classes were heavily unbalanced. Table 1 shows the full predictor set values for all complete cases, each class, and class comparisons. Overall, predictor differences between groups were significant and expected, including, for instance, the higher proportion of current smokers in survivors compared to non-survivors.

**Table 1:** Predictors for all cases, by each class, and univariate class comparisons

Predictors (*n* = 39)	All cases (*n* = 51,943)	Survivors (*n* = 46,233)	Non-survivors (*n* = 5710)	Survivors vs. Non-survivors (*P*)
Age (yrs)	68.8 ± 12.3	67.5 ± 11.9	79.3 ± 9.8	< 0.0001
Male sex	33,620 (64.7)	30,523 (66.0)	3097 (54.2)	< 0.0001
Weight (kg)	79.1 ± 15.9	72.1 ± 15.6	80.0 ± 16.3	< 0.0001
Ambulance to CCU	31,654 (60.9)	27,816 (60.2)	3838 (67.2)	< 0.0001
Comorbid conditions				
Smoking	12,717 (24.5)	11,740 (25.4)	977 (17.1)	< 0.0001
Diabetes	8552 (16.5)	7046 (15.2)	1506 (26.4)	< 0.0001
Hypertension	23,432 (45.1)	20,386 (44.1)	3046 (53.3)	< 0.0001
Previous stroke	3623 (7.0)	2759 (6.0)	864 (15.1)	< 0.0001
Admission medication				
ACE inhibitors	8409 (16.2)	7096 (15.3)	1313 (23.0)	< 0.0001
A2 blockers	5893 (11.3)	5164 (11.2)	729 (12.8)	0.0003
Beta blockers	14,485 (27.9)	12,084 (26.1)	2401 (42.0)	< 0.0001
Statins	9904 (19.1)	8677 (18.8)	1227 (21.5)	< 0.0001
Presenting symptoms				
Chest pain	44,589 (85.8)	40,761 (88.2)	3828 (67.0)	< 0.0001
Dyspnea	3472 (6.7)	2368 (5.1)	1104 (19.3)	< 0.0001
Other	3580 (6.9)	2834 (6.1)	746 (13.1)	< 0.0001
ECG rhythm at CCU				
Sinus	46,297 (89.1)	41,983 (90.8)	4314 (75.6)	< 0.0001
Atrial fibrillation	4469 (8.6)	3308 (7.2)	1161 (20.3)	< 0.0001
ECG QRS at CCU				
Normal	35,819 (69.0)	32,709 (70.7)	3110 (54.5)	< 0.0001
Pathological Q-wave	5407 (10.4)	4753 (10.3)	654 (11.5)	0.0062
Left bundle branch block	2458 (4.7)	1877 (4.1)	581 (10.2)	< 0.0001
Other	5711 (11.0)	4862 (10.5)	849 (14.9)	< 0.0001
ECG STT at CCU				
Normal	11,729 (22.6)	10,805 (23.4)	924 (16.2)	< 0.0001
ST-elevation	17,641 (34.0)	16,251 (35.2)	1390 (24.3)	< 0.0001
ST-depression	11,462 (22.1)	9690 (21.0)	1772 (31.0)	< 0.0001
Other	5820 (11.2)	4726 (10.2)	1094 (19.2)	< 0.0001
Pulmonary rales at CCU				
No	46,205 (89.0)	42,081 (91.0)	4124 (72.2)	< 0.0001
Rales	3880 (7.5)	2762 (6.0)	1118 (19.6)	< 0.0001
Other measures at CCU				
Troponin (ng)	1360 (310–6460)	1350 (280–1587)	1400 (319–10,000)	0.1761
HR(bpm)	76 (65–90)	75 (65–90)	86 (71–86)	< 0.0001
SBP (mm Hg)	148.9 ± 28.6	143.0 ± 28.2	150.0 ± 30.7	< 0.0001
Reperfusion at CCU				
No	34,469 (66.4)	29,740 (64.3)	4729 (82.8)	< 0.0001
Primary PCI	14,665 (28.2)	13,884 (30.0)	781 (13.7)	< 0.0001
Discharge medication				
ACE inhibitors	31,547 (60.7)	28,712 (62.1)	2835 (49.6)	< 0.0001
A2 blockers	6445 (12.4)	5670 (12.3)	775 (13.6)	0.0046
Oral anticoagulants	2993 (5.8)	2514 (5.4)	479 (8.4)	< 0.0001
Other antiplatelet	41,741 (80.4)	38,461 (83.2)	3280 (57.4)	< 0.0001
Beta blockers	46,623 (89.8)	41,789 (90.4)	4834 (84.7)	< 0.0001
Statins	45,366 (87.3)	41,918 (90.7)	3448 (60.4)	< 0.0001
ECG rhythm at discharge				
Atrial fibrillation	3703 (7.1)	2645 (5.7)	1058 (18.5)	< 0.0001

Values are mean ± SD or median (IQR) or count (%). Uncorrected *P*-values are from Welch’s t-tests if variable is Gaussian, Mann-Whitney *U*-tests if non-Gaussian, or Pearson’s χ^2^-tests if categorical

*ACE* angiotensin-converting-enzyme, *A2* angiotensin-2 receptor, *CCU* coronary care unit, *ECG* electrocardiogram, *HR* heart rate, *PCI* percutaneous coronary intervention, *SBP* systolic blood pressure

**Fig. 1:**
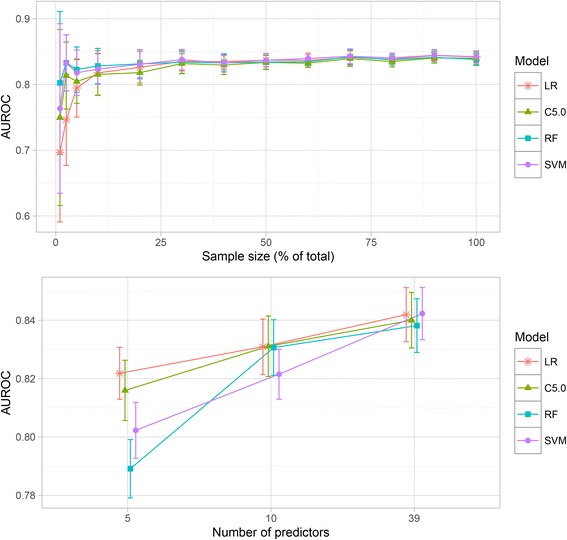
Training results. Top panel: Model training result as a function of increasing sample size (1–100%). Bottom panel: Model training performance on the three predictor sets using 100% of training samples (*n* = 31,166) with the 5 and 10 predictor sets as chosen by each model. Points are mean values of each model’s resampled training runs optimized on the Area Under the Receiver Operating Characteristic (AUROC). *Error bars* indicate ± SD. C5.0, Boosted C5.0; LR, Logistic regression; RF, Random Forest; SVM, Support Vector Machine

Next, we separately evaluated model training with (a) increasing sample size 1–100%, and (b) the three predictor sets. The top panel of Fig. 1 shows how performance increased and stabilised when models were trained on more samples (with number of predictors kept constant at 39). For all models, the training result levelled off using 30% of training data (*n* = 9373, AUROC range ~ 0.82–0.84) providing small gains in robustness and classification acuity thereafter. The bottom panel of Fig. 1 shows that model performance improved when the number of predictors increased (with sample size kept constant at 100%). All models converged with both increasing sample size and increasing number of predictors.

**Fig. 2:**
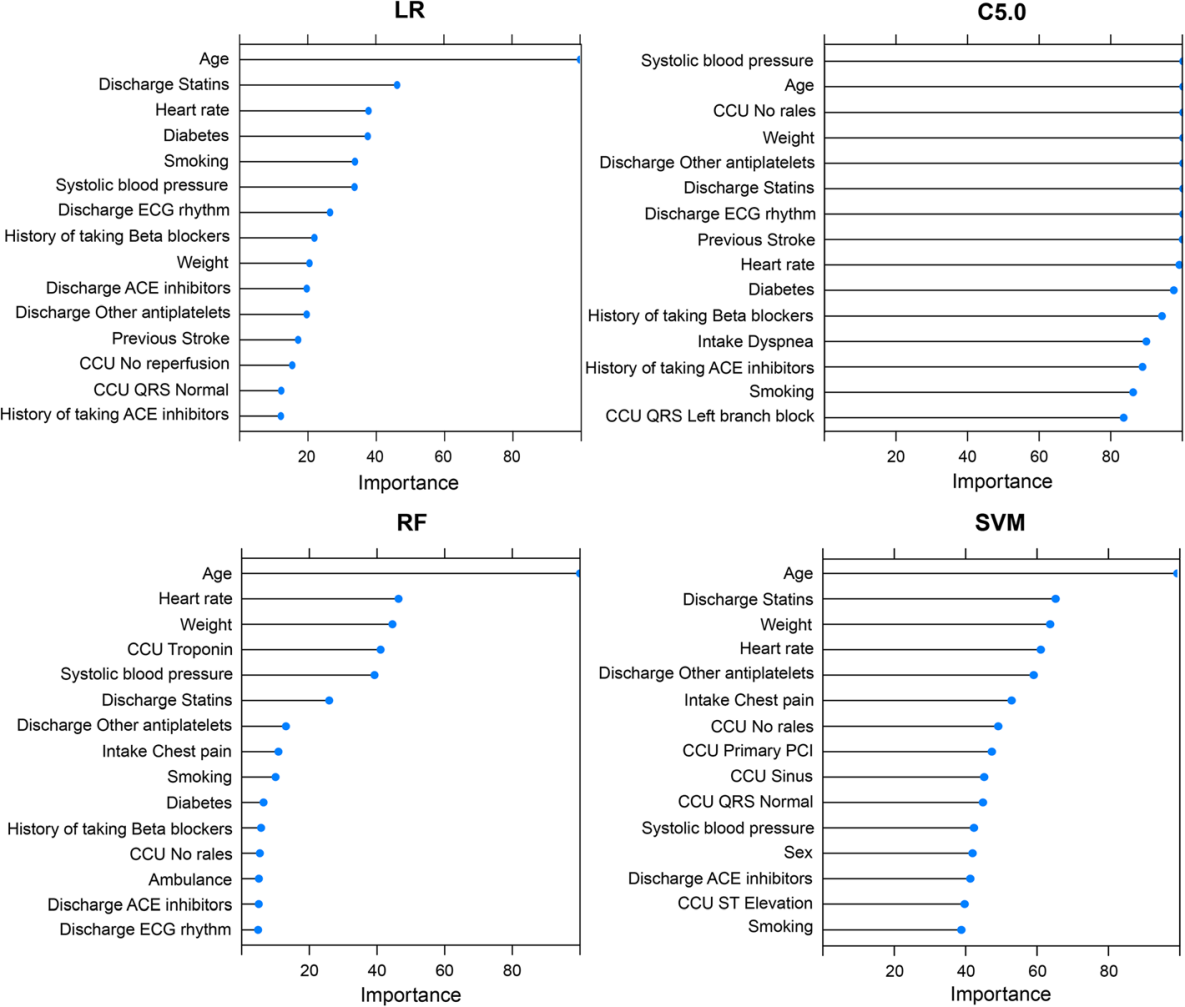
The importance of the 15 most important predictors chosen by each model. Derived from 100% of training samples (*n* = 31,166). Importance is scaled relative to the most important predictor within each model based on model-specific metrics (LR, z-value; C5.0, tree split usage; RF, Gini importance; SVM, univariate AUROC). Prefixes: Previous = before the first MI; Intake = at hospital/lab arrival; CCU = during the Coronary Care Unit stay; Discharge = at discharge from hospital. Unspecified prefix signifies either a fixed predictor or that the predictor was register at some time-point before hospital discharge. C5.0, Boosted C5.0; LR, Logistic regression; RF, Random Forest; SVM, Support Vector Machine; ACE, Angiotensin-converting-enzyme; ECG, Electrocardiogram; PCI, Percutaneous coronary intervention

The importance of the 15 most important predictors as chosen by each model out of the total 39 predictors is displayed in Fig. 2. Some predictors were important for all models (e.g. Age, Statins at discharge, HR), while others were model specific (e.g. Troponin, PCI). Overall, the different models selected a heterogeneous set of most important predictors (cardiac variables, medications, demographics and other).

For the main analysis, we evaluated how trained models predicted the 20,777 complete cases in the untouched test set. Results confirmed our hypothesis that all models performed substantially better than random (AUROC >.70). Comparing models developed on the full 39 predictor set, SVM had the highest test performance (0.845), yet only performed slightly better than C5.0 (vs. 0.841, *P* = 0.0282). LR (0.843) and RF (0.842) performed very similar to SVM and comparisons were non-significant. For the reduced 10 predictor set, C5.0 had the highest test performance (0.834), significantly better than both RF (vs. 0.825, *P* < 0.0001), and SVM (vs. 0.821, *P* < 0.0001). LR (0.830) also performed better than SVM (vs. 0.821, *P* = 0.0103), while remaining comparisons were non-significant. For the minimal 5 predictor set, LR had the highest test performance (0.822) significantly outperforming C5.0 (vs. 0.815, *P* = 0.0134), RF (vs. 0.795, *P* < 0.0001), and SVM (vs. 0.805, *P* < 0.0001). In addition, C5.0 performed significantly better than both RF (*P* < 0.0001) and SVM (*P* = 0.0014), and SVM performed significantly better than RF (*P* = 0.0195). See Table 2 for additional test results.

**Table 2:** Additional test performance metrics

Model	Sens/Spec	PPV/NPV	Detection rate	Detection incidence	Accuracy (95% CI)
Full predictor set (*n* = 39)					
LR	0.771/0.770	0.293/0.965	0.085	0.290	0.770 (0.764 to 0.776)
C5.0	0.798/0.739	0.275/0.967	0.088	0.320	0.746 (0.740 to 0.752)
RF	0.789/0.752	0.282/0.966	0.087	0.307	0.756 (0.750 to 0.762)
SVM	0.784/0.751	0.280/0.966	0.086	0.308	0.755 (0.749 to 0.761)
Reduced predictor set (*n* = 10)					
LR	0.754/0.758	0.278/0.961	0.083	0.298	0.758 (0.752 to 0.763)
C5.0	0.768/0.757	0.281/0.964	0.084	0.301	0.758 (0.752 to 0.764)
RF	0.771/0.746	0.272/0.963	0.085	0.311	0.748 (0.742 to 0.754)
SVM	0.751/0.756	0.275/0.961	0.083	0.300	0.755 (0.749 to 0.761)
Minimal predictor set (*n* = 5)					
LR	0.749/0.750	0.270/0.960	0.082	0.305	0.750 (0.744 to 0.756)
C5.0	0.758/0.736	0.262/0.961	0.083	0.319	0.738 (0.732 to 0.744)
RF	0.755/0.703	0.239/0.959	0.083	0.348	0.708 (0.702 to 0.715)
SVM	0.732/0.753	0.268/0.958	0.080	0.300	0.751 (0.745 to 0.757)

Results of trained models on 100% of testing data (*n* = 20,777) by predictor set. For all models, Base Rate Incidence = 0.110, and No Information Rate = 0.890

*Sens* sensitivity, *Spec* specificity, *PPV* positive predictive value, *NPV* negative predictive value, *CI* confidence interval, *NIR* no information rate, *C5.0* C5.0 boosted decision trees, *LR* logistic regression, *RF* random forest, *SVM* support vector machine

Of the test set 2284 first MI patients found deceased 2 years later, the highest scoring model (SVM, 39 predictors) classified 1791 correctly and 493 incorrectly at the time of hospital discharge. This model also classified 13,894 survivors correctly and 4599 incorrectly. The predictive information gain from hypothetically using this model when also taking into account base incidence rates (column 3 of Table 2) is illustrated by the following example: Before running this model, the average base risk of a patient being deceased 2 years later is 11.0%. If running this model and it indicates survival, the average risk is reduced to 3.4% (NPV). If the model instead indicates non-survival, the average risk of a patient being deceased is increased to 28.0% (PPV). This corresponds to an average 8.2 risk ratio for the outcome in patients classified as non-survivors versus patients classified as survivors.

Modelling was then repeated after adding 27,926 imputed first MI cases (n total = 79,869). This sensitivity analysis showed that models developed on this extended training set (*n* = 47,922) predicted the extended hold-out test set (*n* = 31,947) both similarly between models and slightly better than the primary analyses of complete cases. However, there were some specific model differences. For the 39 predictor set, C5.0 had the highest AUROC (0.879), statistically outperforming RF (vs. 0.875, *P* = 0.0003), SVM (vs. 0.876, *P* = 0.0438), and LR (0.874, *P* < 0.0001), while remaining comparisons were non-significant. Using 10 predictors, C5.0 again performed highest (0.863), significantly higher than LR (vs. 0.858, *P* = 0.0126), and SVM (vs. 0.845, *P* < 0.0001), but not RF (vs. 0.863). In turn, RF performed higher than LR (*P* < 0.0001), and SVM (*P* < 0.0001), and LR performed higher than SVM (*P* < 0.0001). When trained on 5 predictors, LR performed highest (0.851), significantly higher than C5.0 (0.842, *P* < 0.0001), RF (vs. 0.805, *P* < 0.0001), and SVM (0.8303, *P* < 0.0001). Additionally, C5.0 outperformed both RF (*P* < 0.0001), and SVM (*P* < 0.0001), and SVM outperformed RF (*P* < 0.0001). Including the imputed cases, the average base incidence risk was 13.9%. Hypothetically running the best performing model (C5.0, 39 predictors) with one new patient at the time of first MI and the model indicates survival, the average two-year mortality risk is reduced to 4.7% (NPV). If the model instead suggests non-survival the risk increases to 44.2% (PPV). On average, the risk ratio is 9.4 for the outcome in patients classified as non-survivors versus patients classified as survivors by this model.

## Discussion

We used 6 years of high-quality population data from the SWEDEHEART/RIKS-HIA national quality register to evaluate four supervised machine learning algorithms on the unbalanced classification problem of differentiating survivors and non-survivors 2 years after their first MI. When optimized on the *c*-statistic (AUROC), the four trained models showed high and similar performance on the untouched test set. The performance of all models also improved with both increasing sample size and number of predictors. Model training results converged with increasing sample size, especially when 30% or more of the data were used. Adding the remaining 70% of data resulted in modest performance and robustness gains, and practically identical performance regardless of algorithm type.

Regarding the clear performance convergence across models with more samples, we note that a majority of similar ML studies have used fewer samples than 30% of those used herein. Models are generally more variable and unreliable when developed with fewer samples. The present large-scale study therefore highlights a potential problem of data shortage for robust development and performance evaluation of different algorithms. The present study underscores the importance of thorough resampling to counter overfitting, and the need to evaluate predictive models on a hold-out test set exempt from model training. We think that this further highlights the potential problem of data shortage because adequate resampling and data partitioning procedures are intrinsic to building robust models and data shortage hampers both. With that stated, more data is not always better, and factors such as the error in measured values, nature of missing values, data availability, coverage, intended use of constructed models, and more must be considered to arrive at a model’s worth.

### Clinical implications

The 39 predictor SVM showed substantial predictive power for both survivors and non-survivors. Patients classified as survivors by this model had almost a threefold reduction in their base rate risk of dying within 2 years after their first MI discharge. Conversely, patients classified as non-survivors had an almost threefold increase in their base rate risk. The cost of misclassifying true positives versus true negatives is related to what is done differently for patients as a result of classification. In a clinical setting, a positive classification might suggest more monitoring and interventions – which would likely also benefit misclassified survivors. However, the high age of non-survivors puts a natural cap on the possible longevity for these patients. Today, patient mortality risk post MI can be estimated with established risk models such as GRACE [11]. This may not be sufficient, given that the classification (a) performance of models in this study was high, (b) data is continuously collected as part of the clinical routine at all CCUs in Sweden, (c) procedure for a new patient can be almost fully automated with future linkage of the registers to prediction models, (d) could provide decision support for tailored care through patient risk grouping, and (e) could strengthen risk prediction and communication with individual patients. Patient awareness of risk might also be a motivator to make behavioural changes, and clinicians could better target limited resources.

Speculating on how these results might be implemented in clinical practice, we think that one important evaluation would involve a trial design, with clinicians randomized to either predictive modelling support or current practice. Evaluation would then be on the resulting tailored care and clinical outcomes. A health economic analysis of costs would also be beneficial. The objective must be improved care and/or improved targeting of healthcare resources. The current limited routine use of established risk scores would likely benefit from an increased use of improved prediction models for clinical decision making.

Regarding ethics, cardiologists and allied health professionals do not often communicate mortality risk to their patients. Instead it is common to convey the risk for any serious adverse outcome and how that risk can be reduced. The mortality risk is, however, what many prediction models are developed to estimate, and mortality risk is more deliberately discussed between clinicians. In that sense, we see no additional ethical issues. There is however an ethical concern regarding unintended use of these classifiers. Trained models should not be used to differentiate survivors from non-survivors for any other reason than improving healthcare. As with all technology, the responsibility falls on those that approve, develop, implement, and operate it.

### Limitations/Strengths

Missing values are always limiting and results based solely on complete cases can be biased. In the present study, the high completeness of the SWEDEHEART/RIKS-HIA and the Cause of Death register alleviated much of this problem, and was also supported by secondary results after imputation. For the latter, it is important to keep in mind the potential biasing of data due to the imputation procedure. Another possible limitation was that we narrowed the study population to first MI patients, so these models are not useable for recurrent MI patients. Regarding specificity, the 21.6% false positives leaves considerable room for improvement. Reducing this error should be prioritised in future research. Another limitation was the choice of a single outcome. There are other important outcomes, for instance CVD-specific mortality, and other outcome timespans. However, our primary aim was to compare different models on this unique data and selected one of the most important post-MI outcomes for this purpose. The comparison of three popular machine learning algorithms with LR showed that “plain” LR performed similarly to – and sometimes even slightly better than – the more computationally advanced algorithms. This was somewhat surprising, especially since LR does not have any tuning parameters and we did not model any interaction effects. If we assume no data shortage and also account for computation time and model transparency, the faster and more informative LR seems to be “the winner” of the present four-algorithm contest. A slight limitation was inherent in comparing the 10 and 5 predictor models that by design rank predictor importance differently. The main strengths of our study are (1) the first-time evaluation of four popular algorithms on high-quality unselected population data for this clinically relevant classification problem, and (2) the comprehensive set of SWEDEHEART/RIKS-HIA predictors showing that it is possible to build thorough mortality classifiers using many heterogeneous clinical variables that are routinely registered and familiar to clinicians. In addition, we also evaluated three different predictor sets and the influence of sample size on model performance. The present models are by the nature of SWEDEHEART/RIKS-HIA data substantially more generalizable to new first MI patients than previously developed models, and might be useful as complementary decision support tools for improving patient health.

### Future research

Modern medicine has a formidable track-record of applying new technology for identifying and curing disease, prolonging life and improving the quality of life. This has led to a drastic increase in the amount and complexity of patient data. The task of adequately processing and using this information is becoming increasingly unmanageable by humans alone. Our study shows the possibilities of predictive modelling using comprehensive data from high-quality population registers, which should be further investigated in future studies. Several options should be pursued. Instead of imputing missing values, another possibility is to model missing values as potential information, i.e. as additional dummy variables per predictor. In addition, models were highly correlated (*r* > .90) which refrained us from combining them in a meta-ensemble architecture. On the other hand, these imperfect model correlations suggests that a simple linear combination of LR, C5.0, RF, and SVM predictions could yield slightly higher performance than the best single model [35]. We also aim to evaluate deep learning, [36] a logical second step with many samples of complex data. Over time, transfer learning procedures might prove particularly useful. Compared to grid or random search, more advanced techniques such as Bayesian optimization will also be evaluated to attain better tuning settings for model hyperparameters [37]. The prospectively collected and annually updated SWEDEHEART/RIKS-HIA register can provide regular data updates allowing for continuous external model validation and improvement using the most recent high-quality data. The high similarity in first MI patients themselves, their acute MI care, and mortality outcomes in the western World suggests that external model validation outside of Sweden might be possible and potentially beneficial for non-Swedish patients. On the other hand, we should then expect at least some drop in model performance, and if predictors are too different, international validation might be unfeasible. Ultimately this is an empirical question for which external data is needed. It would also be interesting to develop similar models using pooled data from several national population registers.

## Conclusions

Improved risk prediction of two-year mortality at hospital discharge after first myocardial infarction is important for identifying high-risk individuals who may benefit from intensified treatment and care. The performance was high and similar across the four compared models, and because of the superior national coverage, the best model can potentially be used to better differentiate new patients, allowing for improved targeting of limited clinical resources. Using more cases and predictors than most previous studies, model performance converged to the point of algorithm indifference, suggesting insufficient emphasis on data quantity. Future research should focus on further model development and implementation.

## Additional files

Additional file 1: Extended algorithm description and formulae. Abbreviations. Appendix references. (DOCX 26 kb)
