# Supplementary material for: Predicting two-year survival versus non-survival after first myocardial infarction using machine learning and Swedish national register data
Source: BMC Med Inform Decis Mak. 2017 Jul 5;17:99. doi: 10.1186/s12911-017-0500-y (PMC5499032; doi:10.1186/s12911-017-0500-y)
Supplement: Supplementary file 1 — Extended algorithm description and formulae. Abbreviations. Appendix references. (DOCX 26 kb) [file 12911_2017_500_MOESM1_ESM.docx]

Additional file 1

Supplementary to the paper

*Predicting Two-Year Survival Versus Non-Survival After First Myocardial Infarction Using Machine Learning and Swedish National Register Data*

by

*John Wallert, MSc; Mattia Tomasoni, MSc; Guy Madison, PhD; Claes Held, MD, PhD*

in

*BMC Medical Informatics and Decision Making*

1. Extended description and algorithm formulae

*1.1 Binomial Logistic Regression (LR)*

Assuming a Bernoulli distribution of the dependent outcome $(y)$that is conditional on a set of input predictors ${(x}_{1},\ldots,x_{k})$ we can write$y | x_{1},\ldots,x_{k} \sim\mathrm{Bernoulli}(p)$. LR[1] then estimates the binary response probability (Eq. 1) through the function

(1)

$$\log[\frac{pr(y=1|x)}{\left( 1-pr(y=1|x \right)}]=\beta_{0}+ \beta_{1}x_{1}+\ldots{+ \beta}_{k}x_{k}$$

where $(\beta_{0})$ is the intercept and $(\beta_{1},\ldots,\beta_{k})$ are the estimated coefficients. We used LR to generate individual predictive probabilities between 0 and 1 using a cut-off at 0.5 for binary classification. LR lacks tuning parameters, which sets it apart from the other models. Our aim was to benchmark more recently developed models with “out-of-the-box” LR and did not tweak it further. Neither did we include interaction terms to account for possible log non-linear relationships between predictors and the outcome because comparison models were not allowed manual specification at this level of detail.

*1.2 Boosted C5.0 (C5.0)*

Quinlan’s boosted C5.0[2, 3] constructs an ensemble tree through stage-wise development of many decision trees or corresponding rule-sets, emphasizing misclassified cases in previously developed trees. Let ($m)$ denote the classified cases. For the growth of one tree based on these cases $(T_{m})$, the algorithm first decides the predictor and predictor cut-off value that provides the optimal single-split. This decision is based on entropy $(I_{E})$, which is defined

(2)

$$I_{E}\left( f \right)= - \sum_{i=1}^{m} f_{i}{log}_{2}f_{i}$$

Here (Eq. 2) $f_{i}$ denotes the probability of each case being chosen for the split. The greatest reduction in entropy before and after this split is the greatest increase in information gain, since information gain = entropy(before split) – entropy(weighted sum after split), which is analogous to the Kullback-Leibler distance.[4] This first split then results in two branches containing subsets of the cases ${(m}_{1},m_{2})$ for which different predictors and/or predictor cut-offs are used in recursive splits until the branches of $T_{m}$ are fully developed (ends in terminal nodes). C5.0 uses a pessimistic pruning procedure for reducing the tree complexity.[5] After pruning of the first tree, initial weights are calculated and recalibrated for subsequently constructed trees. The weights emphasize misclassified cases in previous trees. The ensemble of trees then majority votes on outcome class. C5.0 boosting is similar to how the AdaBoost algorithm[6] works. C5.0 was tuned over the number of boosting iterations and a logical toggling predictor selection (winnowing/no winnowing).

*1.3 Random Forest (RF)*

Breiman and Cutler´s RF model combines the ideas of bootstrap aggregation and stochastic predictor selection.[7, 8] First, a replacement resampled random subset of cases $(m)$ is generated and one unpruned Classification And Regression Tree (CART)[9] is grown $(T_{m})$ to this subset, where at each node of the tree:

1. A random subset of predictors $(x)$ is selected from the full predictor set ${(x}_{1},\ldots,x_{k})$
2. Based on $(x)$, the optimal split is decided using Gini impurity, computed as

(3)

$$I_{G}\left( f \right)= \sum_{i=1}^{m} f_{i}(1- f_{i})= \sum_{i=1}^{m} \left( f_{i}- f_{i}^{2} \right)= \sum_{i=1}^{m} f_{i}- \sum_{i=1}^{m} f_{i}^{2}=1- \sum_{i=1}^{m} f_{i}^{2}= \sum_{i\neq k} f_{i}f_{k}$$

Here (Eq. 3) the probability of randomly selecting each case $(f_{i})$ is multiplied by the probability of each case being misclassified$(1- f_{i})$. When all cases in the node fall into one category, Gini impurity is zero. Many such trees are then grown uncorrelated to each other, forming an ensemble${\{T_{m}\}}_{1}^{M}$. For classification of an unseen data point$(u)$, denote the class prediction of the *m*th tree$\hat{C}_{m}(u)$. The ensemble then predicts

(4)

$$\hat{C}_{rf}^{M}\left( u \right)=majority vote {\{\hat{C}_{m}\left( u \right)\}}_{1}^{M}$$

Thus, like C5.0, the RF ensemble of trees majority votes on class (Eq. 4). RF was tuned over the number of randomly chosen predictors (mtry).

*1.4 Support Vector Machine (SVM)*

A SVM[10, 11] creates a multidimensional space where each dimension is mapped to one of the predictors $(x)$. Each individual case is represented by a point $(u)$ in this multidimensional space, labelled as belonging to some class: either survivors or non-survivors, in our case. SVMs are trained by automatically fitting a separating function (kernel) through multidimensional space to maximize the separation margin between the two classes, using the support from individual points closest to each other but with opposite labels (the support vectors). To better suit the data, the kernel used as a divide between the classes can be chosen among a number of functions. In addition, a number of function-specific parameters can be tuned (e.g. the steepness in the case of a linear kernel, the radius in the case of a spherical kernel). For this study we applied a soft-margin SVM with a non-linear radial basis kernel with the slack variable (cost function) as tuning parameter. Tuning the cost function means varying the degree of allowed spatial overlap between classes.

Assume binary classes are coded -1 or 1. The SVM estimates $n$ parameters for the prediction function, and for a new point $u$ in multidimensional space, the SVM predicts

(5)

$$f\left( u \right)=\beta_{0}+\sum_{i=1}^{n} a_{i}y_{i}x_{i}^{'}u$$

This (Eq. 5) is a function of the inner product of vectors which allows for expanding the predictor space with a kernel function $K(x, y)$. In the present case, the radial basis function (Eq. 6).

(6)

$$K(x, u)=\exp[\frac{-\sigma}{2}{(x-u)}^{2}]$$

The decision process for the unseen point ($u)$ is then: If $f\left( u \right)>0$ class 1 is predicted, otherwise class -1 is predicted. Since SVMs do not naturally produce predictive probabilities, Platt´s algorithm was used to fit a logistic function to the raw SVM class prediction scores $f(u_{k})$, transforming them using two maximum-likelihood estimated scalars $\left( A, B \right)$ according to (Eq. 7).

(7)

$$pr\left( y=1 | u_{k} \right)= \frac{1}{1+exp(Af\left( u_{k} \right)+B)}$$

2. Abbreviations

A2: Angiotensin-2 Receptor

ACE: Angiotensin Converting Enzyme

AdaBoost: Adaptive Boosting

AUROC: Area Under the Receiver Operating Characteristic Curve

CART: Classification and Regression Trees

C5.0: Boosted C5.0 trees/rule-sets

CCU: Coronary Care Unit

CHD: Coronary Heart Disease

CI: Confidence Interval

CVD: Cardiovascular Disease

ECG: Electrocardiogram

GRACE: Global Registry of Acute Coronary Events

HR: Heart Rate

ICD: International Statistical Classification of Diseases and Related Health Problems

Kg: Kilogram

LR: Logistic Regression

MI: Myocardial Infarction

ML: Machine Learning

NIR: No Information Rate

NPV: Negative Predictive Value

PCI: Percutaneous Coronary Intervention

PPV: Positive Predictive Value

Preval: Prevalence

RF: Random Forest

RIKS-HIA: Register for Information and Knowledge about Swedish Heart Intensive Care Admissions

SBP: Systolic Blood Pressure

Sens: Sensitivity

Spec: Specificity

SVM: Support Vector Machine

SWEDEHEART: Swedish Web-system for Enhancement and Development of Evidence-based care in Heart disease Evaluated According to Recommended Therapies

U-CARE: Uppsala University Psychosocial Care Programme

3. Appendix references

1. Cox DR. The Regression Analysis of Binary Sequences. J R Stat Soc Series B. 1958;20:215-42. URL: <https://www.jstor.org/stable/2983890?seq=1#page_scan_tab_contents>. Accessed 29 June 2016.

2. Quinlan JR. C4.5: Programs for Machine Learning. Morgan Kaufmann Publishers, 1993.

3. Kuhn M, Weston S, Coulter N, Culp M and Quinlan R. C5.0 Decision Trees and Rule-Based Models. 0.1.0-24 ed. CRAN2015. URL: <https://cran.r-project.org/web/packages/C50/C50.pdf>. Accessed 1 September 2016.

4. Kullback S and Leibler RA. On Information and Sufficiency. Ann Math Statist. 1951;22:79-86. URL: <https://projecteuclid.org/download/pdf_1/euclid.aoms/1177729694>. Accessed 1 September 2016.

5. Wu X, Kumar V, Ross Quinlan J, et al. Top 10 algorithms in data mining. Knowl Inf Sys. 2007;14:1-37. URL: <http://www.cs.uvm.edu/~icdm/algorithms/10Algorithms-08.pdf>. Accessed 1 September 2016.

6. Freund Y and Schapire RE. A Decision-Theoretic Generalization of On-Line Learning and an Application of Boosting. J Comput Syst Sci. 1997;55:119-39. URL: <http://www.sciencedirect.com/science/article/pii/S002200009791504X>. Accessed 1 September 2016.

7. Ho TK. Random Decision Forests. Third International Conference on Document Analysis and Recognition. Montreal, QC1995, p. 278-82. URL: <http://ect.bell-labs.com/who/tkh/publications/papers/odt.pdf>. Accessed 1 September 2016.

8. Breiman L. Random Forests. Machine Learning. 2001;45:5-32. URL: <http://link.springer.com/article/10.1023/A:1010933404324>. Accessed 1 September 2016.

9. Breiman L, Friedman J, Olshen R and Stone C. Classification and regression trees. Wadsworth Books, 1984.

10. Boser, B. E.; Guyon, I. M.; Vapnik, V. N. (1992). "A training algorithm for optimal margin classifiers".

Proceedings of the fifth annual workshop on Computational learning theory – COLT '92. p. 144. URL: <http://w.svms.org/training/BOGV92.pdf>. Accessed 1 September 2016.

11. Cortes C and Vapnik VN. Support-Vector Networks. Machine Learning. 1995;20:273-97. URL: [http://image.diku.dk/imagecanon/material/cortes_vapnik95.pdf. Accessed 1 September 2016](http://image.diku.dk/imagecanon/material/cortes_vapnik95.pdf.%20Accessed%201%20September%202016).
